# Temporal and Demographic Variations in Hospital‐Based Influenza A Virus Positivity Rates Before, During, and After the COVID‐19 Pandemic: A 6‐Year Single‐Center Study in Southwest China

**DOI:** 10.1002/iid3.70605

**Published:** 2026-11-10

**Authors:** Hao Dang, Xiujuan Peng, Li Xu, Wei Wang, Ping Tang

**Affiliations:** ^1^ Department of Clinical Laboratory The Third Hospital of Mianyang (The Affiliated Mianyang Hospital of Chongqing Medical University) Mianyang Sichuan China; ^2^ Department of Thyroid, Head, Neck and Maxillofacial Surgery The Third Hospital of Mianyang (The Affiliated Mianyang Hospital of Chongqing Medical University) No.190 The East Jiannan Road Mianyang Sichuan China

**Keywords:** China, COVID‐19, hospital‐based test positivity rates, Influenza A Virus, pandemic, surveillance, testing methods

## Abstract

**Objective:**

To characterize the epidemiological dynamics of hospital‐based influenza A virus (IAV) test positivity rates (TPR) across the pre‐, during‐, and post‐COVID‐19 pandemic periods in Southwest China, while addressing biases introduced by evolving testing methodologies and healthcare‐seeking behaviors.

**Methods:**

A total of 57,704 influenza A test records from a symptom‐driven, care‐seeking population at the Third Hospital of Mianyang were retrospectively analyzed from 2018 to 2024. Detection methods included colloidal gold immunoassay (Stages 1–2) and quantitative PCR (Stage 3). Because assay type was completely confounded with pandemic stage, test‐performance comparisons across modalities were not feasible; all findings are interpreted as hospital‐based TPR rather than community prevalence.

**Results:**

The overall IAV TPR was 14.8%. TPR dropped sharply to 4.5% during the implementation of non‐pharmaceutical interventions (NPIs) and surged to 17.8% in the post‐pandemic period, peaking in 2023. Age‐specific positivity patterns shifted markedly across stages, consistent with a composite of changing viral circulation, testing thresholds, and clinical triage practices.

**Conclusion:**

IAV test positivity rebounded significantly following the relaxation of containment measures. Interpretations of TPR must account for detection‐method biases, variations in testing volume, and clinical triage practices, as TPR is a proxy for healthcare system stress rather than a direct measure of community prevalence. Stage‐specific, methodologically transparent surveillance is warranted to inform future pandemic preparedness.

## 1 Introduction

Influenza is a contagious respiratory disease that leads to annual seasonal epidemics, resulting in approximately 1 billion infections, 3 to 5 million severe cases, and 290,000–650,000 deaths worldwide each year [1]. Among the different types of influenza viruses, influenza A virus (IAV) and influenza B virus (IBV) circulate and cause seasonal epidemics of disease, only IAV has been known to cause pandemics [2]. The severity of these pandemics depends on various factors, including the virulence of the virus strain and the population's existing immunity [3]. The 2009 H1N1 pandemic, the first influenza pandemic in decades, illustrated how IAV can undergo significant antigenic changes, such as antigenic drift and shift, which complicate prevention and control efforts. This underscores the need for continuous surveillance and thorough analysis of infection trends to effectively shape public health strategies.

Understanding the factors that influence IAV infection rates is critical in managing its spread. Various demographic factors, including age [4], presence of chronic illness [5], have been identified as significant determinants of susceptibility to infection and disease severity. Additionally, the timing and context of testing, whether during different stages of an epidemic or in different years, can significantly impact the detection rates and subsequent public health responses [6]. Furthermore, vaccination plays a vital role in this landscape; however, its effectiveness can be influenced by these demographic factors and the timing of administration [7]. Importantly, vaccine efficacy can be significantly compromised in cases of antigenic mismatch between the vaccine and the circulating virus strain. Lastly, the main testing methods used for virus detection, such as polymerase chain reaction (PCR) and colloidal gold testing, vary in sensitivity and specificity, influencing the reported positivity rates [8]. Crucially, reported positivity rates, often expressed as test positivity rates (TPR), are composite metrics influenced not only by viral transmission but also by testing volume, clinical thresholds for test ordering, and the specific diagnostic assays employed.

This study aims to provide a comprehensive analysis of hospital‐based IAV test positivity rates (TPR) in Southwest China from 2018 to 2024, spanning the pre‐pandemic, pandemic, and post‐pandemic phases of COVID‐19. By leveraging a large dataset of 57,704 influenza A test records, we characterize TPR dynamics across different pandemic stages, years, age groups, and detection methods. Recognizing the inherent limitations of facility‐based surveillance, we explicitly address potential biases arising from evolving testing methodologies, clinical triage practices, and the complete confounding between assay type and pandemic stage. Our findings aim to disentangle viral activity from healthcare system factors, thereby providing a more robust evidence base for interpreting surveillance data and optimizing future public health responses.

## 2 Methods

### 2.1 Study Population

This study analyzed the epidemiological patterns of Influenza A across the pre‐, during‐, and post‐pandemic periods. Data were extracted from the Laboratory Information System (LIS) of the Third Hospital of Mianyang (a large tertiary comprehensive hospital in Southwest China and the Affiliated Mianyang Hospital of Chongqing Medical University), covering tests conducted from October 26, 2018, to July 11, 2024. The analytic dataset comprised influenza A test records from individuals undergoing testing across various clinical departments, including outpatients, emergency departments, and inpatient units. Testing practices were predominantly symptom‐driven or severity‐based, prioritizing severe cases and high‐risk groups for PCR confirmation, rather than constituting universal pre‐admission screening. Records lacking key variables (e.g., age, sex, or test result) were excluded. The unit of analysis was the individual test record rather than the individual patient. Because the LIS extract contained no patient‐level identifier, repeat tests from the same individual could not be identified, and the number of such repeat tests was therefore not quantified. The study protocol complied with ethical guidelines and was approved by the Ethics Committee of the Third Hospital of Mianyang.

### 2.2 Data Collection

The data for this study was meticulously collected through a comprehensive process of influenza A testing. Two main test methods were included: colloidal gold immunoassay (antigen detection) and real‐time polymerase chain reaction (PCR) techniques. For PCR testing: Samples (nose and throat swabs) were obtained from a defined patient cohort meeting our inclusion criteria. Sample collection was performed under sterile conditions to prevent contamination and was carried out by well‐trained general practitioners to ensure the integrity of RNA. Throat swabs were taken using sterile swab sticks, ensuring that the swab was in contact with the posterior pharynx and tonsillar areas. Samples were stored in tubes, labeled, coded for anonymity, and transported to the laboratory under controlled conditions (maintained at 2°C–8°C). Upon receipt, samples were assessed for volume and integrity before RNA extraction and PCR processing. For colloidal gold testing: Throat and nose swabs were collected by well‐trained general practitioners using sterile swab sticks, with proper sampling from the posterior pharynx and tonsillar areas (throat) or nasal mucosa (nose) to ensure adequate antigen presence. Swabs were placed in the extraction buffer provided with the kit immediately after collection. Samples were labeled, coded for anonymity, and tested at the point of care or transported to the laboratory under ambient conditions within the manufacturer‐recommended timeframe. Upon receipt, samples were assessed before performing the antigen detection assay.

Detection of influenza A virus was performed using the One Step Influenza A&B Test Kits (Guangzhou Wondfo Biotech Co. Ltd.) for colloidal gold assays and Six Respiratory Tract Pathogen Nucleic Acid Detection Kits (PCR‐fluorescence probe method) (Sansure Biotech Inc.) for PCR testing, according to the manufacturers' protocols.

Data extraction was performed meticulously, with results recorded electronically in a secure database. Each sample's test result was linked to its corresponding patient data. This database was regularly backed up to prevent data loss and was accessible only to authorized personnel involved in the study.

### 2.3 Statistical Analysis

The statistical analysis was tailored to the study design and data structure. Although age was recorded as a continuous variable, it was categorized into predefined groups (e.g., 0–6, 7–17, 18–59, ≥ 60 years) for the primary analysis to align with clinical stratification practices. Descriptive statistics were presented as frequencies and percentages for categorical variables. Medians and interquartile ranges were calculated for continuous measures to summarize central tendency and dispersion.

Comparisons of test‐record characteristics across the three pandemic stages were performed using Pearson's Chi‐square tests for categorical variables. Given the categorical treatment of age, formal normality testing was omitted. Logistic regression models were employed to estimate odds ratios (ORs) and 95% confidence intervals (CIs) for the associations between age groups, sex, testing method, pandemic stage, and Influenza A virus (IAV) positivity. All analyses were conducted using R (version 4.2.3) within the RStudio environment (version 2023.03.0 Build 386), with statistical significance defined as a two‐tailed *p* < 0.05.

Temporal Denominator Imbalance: A critical caveat in interpreting stage‐specific trends is the profound imbalance in testing volume across periods. Monthly testing volumes averaged approximately 670 tests in Stage 1 (October 2018–December 2019), plummeted to ~270 tests in Stage 2 (January 2020–December 2022), and surged to ~2000 tests in Stage 3 (December 2022–July 2024). This near order‐of‐magnitude fluctuation in testing intensity—particularly the exceptionally low throughput during Stage 2—indicates that Stage 2 positivity rates likely reflect a highly selective subgroup (e.g., severe cases or high‐risk exposures) rather than representative community transmission. Consequently, comparisons between Stage 2 and Stage 3 are susceptible to spectrum bias and should be interpreted as reflections of shifting testing priorities and healthcare‐seeking behaviors, rather than unambiguous indicators of viral prevalence.

## 3 Results

Our extensive analysis uncovered substantial fluctuations in Influenza A test positivity rates, which were stratified by detailed age groups (0–6, 7–17, 18–59, > = 60 years), sexes (Male, Female), test methods (Colloidal gold, PCR), and pandemic stages (Pre‐pandemic, During pandemic, Post‐pandemic).

### 3.1 General and Clinical Characteristics of the Study Population

This article summarized the general and clinical characteristics of the study population, comprising 57,704 influenza A test records across three pandemic stages. The distribution of age groups showed substantial variation, with the highest proportion of tests in the 7–17 years category (37.45%). The sex distribution was nearly even, with males slightly outnumbering females (52.5% vs. 47.5%). Testing methods also varied substantially across stages, with colloidal gold being the most common method overall (69.3%), though its use declined in Stage 3 as PCR became more prevalent (46.5%). Test results indicated an overall positivity rate of 14.8%, with the highest rate observed in Stage 3 (17.8%). These findings underscore the demographic shifts and evolving testing strategies throughout the pandemic stages (Table 1).

**Table 1:** General and clinical characteristics of the study population (*n* = 57,704)*.

Characteristics	Overall	Stage 1	Stage 2	Stage 3	*p* value
		(*n* = 10,059)	(*n* = 9502)	(*n* = 38,143)	
Age group					< 0.001
0–6 years	17,194 (29.80%)	4529 (45.0%)	2519 (26.5%)	10,146 (26.6%)	
7–17 years	21,611 (37.45%)	2959 (29.4%)	3471 (36.5%)	15,181 (39.8%)	
18–59 years	12,040 (20.87%)	1873 (18.6%)	2539 (26.7%)	7628 (20.0%)	
> = 60 years	6859 (11.89%)	698 (6.9%)	973 (10.2%)	5188 (13.6%)	
Sex					< 0.001
Female	27,419 (47.5%)	4887 (48.6%)	4341 (45.7%)	18,191 (47.7%)	
Male	30,285 (52.5%)	5172 (51.4%)	5161 (54.3%)	19,952 (52.3%)	
Method					< 0.001
Colloidal gold	39,969 (69.3%)	10,059 (100%)	9502 (100%)	20,408 (53.5%)	
PCR	17,735 (30.7%)	0 (0.0%)	0 (0%)	17,735 (46.5%)	
Result					< 0.001
Negative	49,177 (85.2%)	8748 (87.0%)	9070 (95.5%)	31,359 (82.2%)	
Positive	8527 (14.8%)	1311 (13.0%)	432 (4.5%)	6784 (17.8%)	

*Note:* * Data are presented as *n* (%) for categorical variables. Stage 1 is before 24 January 2020; Stage 2 spans 24 January 2020 to 19 December 2022; and Stage 3 begins on 20 December 2022. The cutoff of 19 December 2022 was chosen to coincide with a clear inflection in local testing dynamics following the release of the “New 10 Measures” (November 2022). Data from our hospital's Laboratory Information System (LIS) show a sharp increase in outpatient respiratory testing volume beginning in mid‐December 2022, with 19 December marking a distinct operational and epidemiological breakpoint. The breakpoint is justified by this testing‐volume inflection alone.

### 3.2 Dynamic Trends in Hospital‐Based IAV Positivity Rates: Stage‐Specific Variations, Sex Differences, and Detection Method Effects

The study's analysis of positivity detection rates for the Influenza A Virus across different stages revealed significant variability influenced by age, sex, and detection methods. The positivity rate was 14.8% across all stages, with notable fluctuations observed throughout the study period. Specifically, the positivity rate was in Stage 1 at 13.0%, dipped to 4.5% in Stage 2, and then rose again to 17.8% in Stage 3 (Table 2 and Figure 1). Figure 1 visualizes this comparison, underscoring the dynamic nature of virus detection rates over time.

**Table 2:** Positivity detection rates and 95% Confidence Intervals of Influenza A virus infection across Stages (*n* = 57,704)*.

	All Stages	Stage 1	Stage 2	Stage 3
		(*n* = 10,059)	(*n* = 9502)	(*n* = 38,143)
Overall	15% [14%, 15%]	13% [12%, 14%]	5% [4%, 5%]	18% [17%, 18%]
Age group				
0–6	18% [17%, 19%]	14% [13%, 16%]	5% [4%, 5%]	22% [21%, 22%]
7–17	14% [13%, 14%]	15% [14%, 16%]	8% [7%, 9%]	16% [16%, 17%]
18–59	14% [13%, 15%]	9% [7%, 10%]	2% [2%, 3%]	18% [17%, 19%]
> = 60	12% [11%, 12%]	9% [7%, 11%]	1% [1%, 2%]	15% [15%, 16%]
*p*‐value	< 0.001	< 0.001	< 0.001	< 0.001
Sex				
Female	14% [13%, 15%]	12% [11%, 13%]	4% [4%, 5%]	17% [17%, 18%]
Male	15% [14%, 15%]	14% [13%, 15%]	5% [4%, 6%]	18% [18%, 19%]
*p*‐value	0.032	0.048	0.062	0.134
Method				
Colloidal gold	NA	13% [12%, 14%]	5% [4%, 5%]	22% [21%, 22%]
PCR	NA	NA	NA	13% [12%, 13%]
*p*‐value	NA	NA	NA	< 0.001

*Note:* *Data are presented as proportions. Stage 1 is before 23 January 2020; stage 2 is from 24 January 2020 to 19 December 2022; and stage 3 is after 19 December 2022.

**Figure 1:**
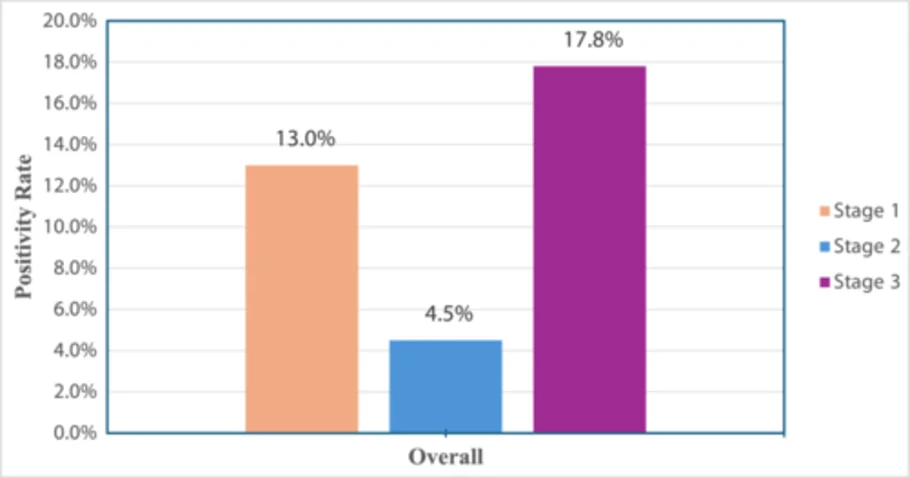
The dynamic nature of hospital‐based test positivity rates (TPR) for Influenza A Virus (IAV) across three pandemic stages of COVID‐19.

Sex‐specific analysis revealed that males exhibited slightly higher positivity rates than females across most stages. This difference was statistically significant in Stage 1 (*p* = 0.048) but not in Stage 2 (*p* = 0.062) or Stage 3 (*p* = 0.134). However, the magnitude of the sex effect was small (e.g., an odds ratio of 1.09 in one cohort and 1.00 in the other), indicating that the observed sex difference carries limited epidemiological weight despite nominal statistical significance. Despite this waning significance in later stages, the overall sex difference remained statistically significant across the entire study period (*p* = 0.032), suggesting a subtle but persistent male predominance in IAV detection within this care‐seeking population. Detection methods also played a critical role, colloidal gold testing yielded a higher positivity rate of 22%, compared to 13% for PCR testing in Stage 3. Nevertheless, this difference should not be construed as evidence of superior sensitivity for colloidal gold. Given that PCR is the established reference standard with higher sensitivity, the observed disparity is more plausibly attributed to clinical triage, where colloidal gold was preferentially used for symptomatic outpatients (high pre‐test probability) while PCR was reserved for complex inpatients.

### 3.3 Positive Detection Counts of Hospital‐Based Influenza A by Year and Methods

The study detailed the number of tests performed and the corresponding positive results from 2018 to 2024. Testing and positive cases surged in 2023, with 26,592 tests performed and 5006 positives, marking the highest count during the study. Although testing slightly decreased in 2024, it still resulted in a substantial number of positives. Early years, like 2018 and 2019, had much lower testing and positive counts, but these steadily increased over time, reflecting improved testing capabilities (Figure 2A).

**Figure 2:**
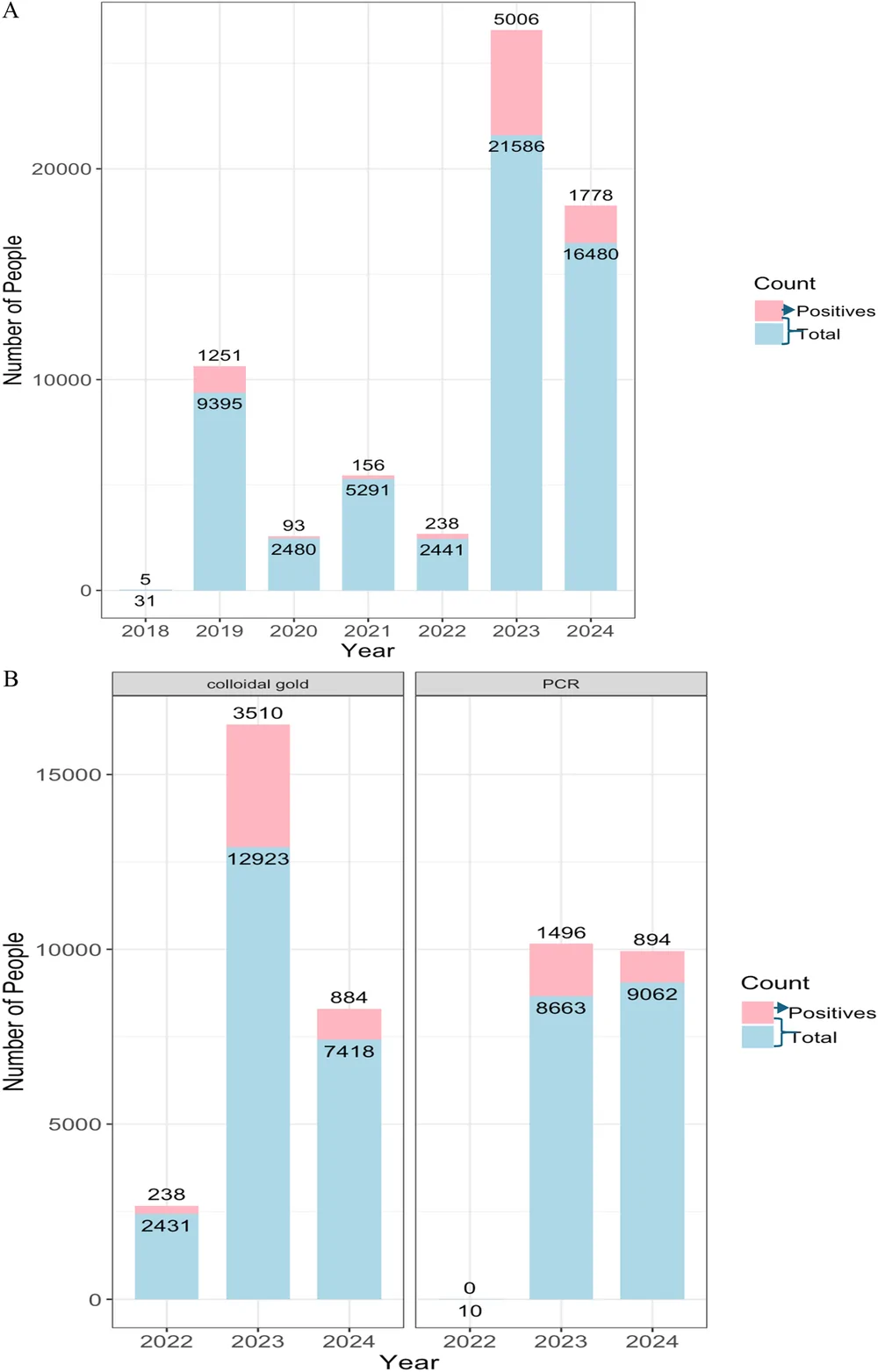
(A) Positive detection counts of hospital‐based test positivity rates (TPR) for Influenza A Virus (IAV) from 2018 to 2024. (B) Annual distribution of hospital‐based test positivity rates (TPR) for Influenza A Virus (IAV) by detection methods, 2022–2024. PCR tests in 2022: denominator = 10. Bars show stacked counts of negative (lower segment) and positive (upper segment) tests, so that bar height equals the total number of tests: in 2023, colloidal gold = 16,433 tests (12,923 negative + 3510 positive) and PCR = 10,159 tests (8663 negative + 1496 positive).

The two methods also differed in positivity rate. In 2023, the positivity rate was 21.4% for colloidal gold (3510/16,433 tests) and 14.7% for PCR (1496/10,159 tests); that is, colloidal gold yielded more positives per 100 tests performed, not merely a larger absolute count (3510 vs. 1496). Because the two assays were applied to different patient streams under clinical triage during the post‐pandemic surge, this difference should not be interpreted as reflecting a difference in analytical sensitivity (Figure 2B).

### 3.4 Positive Detection Rate Trends of Hospital‐Based Influenza A by Year, Month and Methods

The study showed the yearly positivity rates by detection method from 2018 to 2024. Colloidal gold exhibited a declining trend from 2018, reaching a low in 2021, followed by a sharp increase in 2023, peaking at 21.4%. PCR, introduced in 2022, revealed lower positivity rates but also peaked in 2023 at 14.7%, before stabilizing in 2024. The substantial rise in positivity during 2023 suggests a potential outbreak or increased virus activity (Figure 3A).

**Figure 3:**
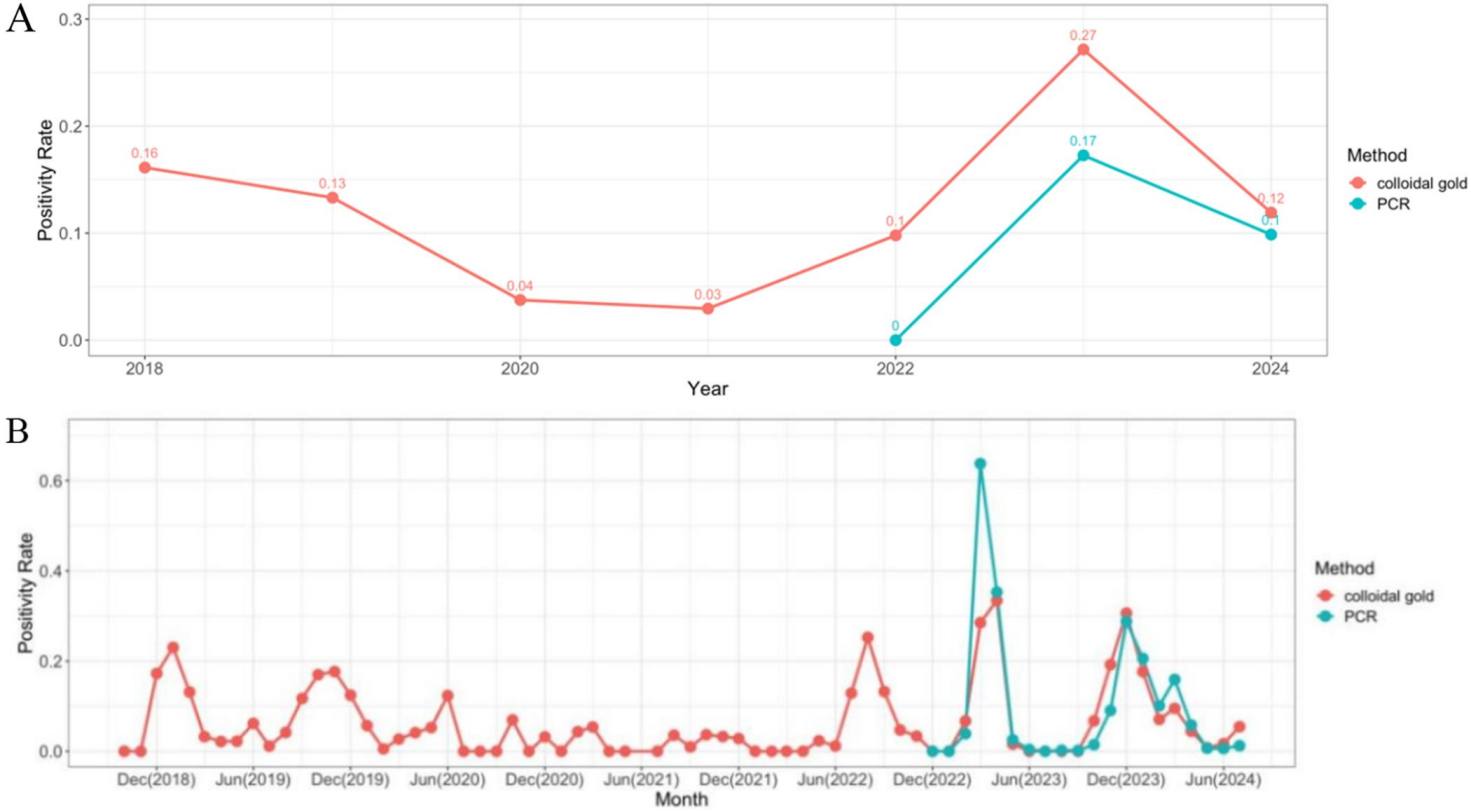
(A) Temporal trends in hospital‐based test positivity rates (TPR) for Influenza A Virus (IAV) by detection method, 2018–2024. PCR tests in 2022: denominator = 10. In 2023, denominators were 16,433 tests for colloidal gold and 10,159 tests for PCR. (B) Temporal trends in test positivity rates (TPR) for IAV from 2018 to 2024. The number of tests contributing to each monthly data point is labeled for the PCR series.

The study also provided monthly positivity rates, showing considerable fluctuation, particularly during late 2022 to mid‐2023, consistent with the yearly trends. Both detection methods displayed similar trends, reinforcing the observation of heightened Influenza A Virus activity during this period (Figure 3B).

### 3.5 Logistic Regression Analysis of Factors Influencing Hospital‐Based Influenza A Virus Test Results

The study presented a logistic regression analysis of factors influencing Influenza A Virus test results from 2018 to 2024 (Table 3). For the colloidal gold method, male sex was associated with slightly increased odds of a positive result (OR = 1.09, 95% CI: 1.03–1.15) compared with female sex. In contrast, among colloidal gold tests, individuals aged 7–17 (OR = 0.74, 95% CI: 0.69–0.79), 18–59 (OR = 0.49, 95% CI: 0.45–0.53), and ≥ 60 years (OR = 0.23, 95% CI: 0.20–0.26) all showed significantly lower odds of testing positive relative to those aged 0–6 years, with the reduction most pronounced in the oldest group. For the PCR cohort, sex was not significantly associated with positivity (male vs. female: OR = 1.00, 95% CI: 0.92–1.09), whereas older age groups (7–17, 18–59, and ≥ 60 years) had significantly higher odds of a positive result compared with the < 7 reference group (OR = 1.87, 2.66, and 2.59, respectively; all *p* < 0.001). Notably, all PCR tests in this study were conducted after 19 December 2022 (Stage 3); therefore, the stage variable was constant for the PCR cohort and not included in its regression model. Within the colloidal gold method, study stage significantly impacted results: Stage 2 was associated with substantially decreased odds of positivity (OR = 0.35, 95% CI: 0.32–0.40), whereas Stage 3 was associated with markedly increased odds (OR = 2.22, 95% CI: 2.07–2.38) relative to Stage 1. These findings underscore the importance of considering age, detection method, and epidemic stage when interpreting test results and making public health decisions.

**Table 3:** Statistics of logistic regression analysis of factors influencing Influenza A Virus infection test results from 2018 to 2024 (*n* = 57,704) *.

Variables	Colloidal Gold method (*n* = 39969)		PCR method (*n* = 17735)	
	OR (95% CI)	*p* value	OR (95% CI)	*p* value
Sex				
Female	Ref.		Ref.	
Male	1.09 (1.03–1.15)	0.003	1.00 (0.92–1.09)	0.980
Age group (years)				
< 7	Ref.		Ref.	
7–17	0.74 (0.69–0.79)	< 0.001	1.87 (1.61–2.19)	< 0.001
18–59	0.49 (0.45–0.53)	< 0.001	2.66 (2.33–3.03)	< 0.001
≥ 60	0.23 (0.20–0.26)	< 0.001	2.59 (2.32–2.89)	< 0.001
Stage				
Stage 1	Ref.		—	—
Stage 2	0.35 (0.32–0.40)	< 0.001	—	—
Stage 3	2.22 (2.07–2.38)	< 0.001	—	—

*Note:* *OR = Odds Ratio; CI = Confidence Interval; Ref. = Reference group. Stage 1 is before 23 January 2020; stage 2 is from 24 January 2020 to 19 December 2022; and stage 3 is after 19 December 2022. Because all PCR tests in this study were conducted after 19 December 2022, the “Stage” variable was a constant (Stage 3) for the PCR cohort and thus not included in the PCR logistic regression model.

## 4 Discussion

This study provides a comprehensive analysis of hospital‐based test positivity rates (TPR) for Influenza A Virus (IAV) across various stages, years, age groups, and detection methods over a 6‐year period, encompassing pre‐pandemic, pandemic, and post‐pandemic phases. The findings reveal significant variations in positivity rates influenced by demographic factors, testing methods, and the timing of the pandemic stages, highlighting critical insights into the epidemiology of IAV.

The analysis indicates that temporal trends in TPR fluctuated significantly across the different stages. The highest TPR was observed during Stage 3 (post‐pandemic period). While this likely reflects a genuine resurgence in community viral activity following the relaxation of COVID‐19 restrictions, the magnitude of this increase must be interpreted cautiously. A rise in TPR can occur when overall testing volumes fall and clinical thresholds for testing tighten, leading to a higher proportion of tests being performed on severe or high‐risk cases. Conversely, the lowest TPR during Stage 2 coincided with the peak of the COVID‐19 pandemic. Widespread public health measures (e.g., social distancing, mask‐wearing) likely suppressed both virus circulation and altered testing behaviors [9, 10, 11, 12, 13].

Demographically, age emerged as a critical factor influencing positivity rates, albeit with distinct patterns depending on the detection method employed. MultIVAriable logistic regression (Table 3) revealed a stark contrast between testing modalities. For the colloidal gold method, younger children (0–6 years) exhibited the highest odds of testing positive. Compared to this reference group, older individuals showed significantly lower odds, with the reduction becoming more pronounced with advancing age (7–17 years: OR = 0.74; 18–59 years: OR = 0.49; ≥ 60 years: OR = 0.23). Conversely, within the PCR cohort, which exclusively comprised samples from Stage 3, older age groups were associated with significantly higher odds of positivity compared to the 0–6‐year reference (7–17 years: OR = 1.87; 18–59 years: OR = 2.66; ≥ 60 years: OR = 2.59). These findings align with existing literature suggesting that while young children are physiologically more susceptible to infection [14, 15] and often have lower vaccination rates [16, 17, 18], the observed age distribution in PCR testing likely reflects differential healthcare‐seeking behaviors and testing priorities during the post‐pandemic surge, where older adults and symptomatic individuals were more likely to undergo confirmatory PCR testing. Furthermore, older adults (≥ 60 years) displaying the lowest positivity in the colloidal gold analysis may reflect higher vaccination coverage or reduced community exposure during restrictive phases. Fortunately, it is reported that the promotion of blanket vaccination has brought major reductions in morbidity and mortality [19].

Regarding detection methodologies, it is crucial to acknowledge a significant limitation: the testing method was almost completely confounded with the pandemic stage. Colloidal gold testing was the sole modality utilized during Stages 1 and 2, whereas PCR testing was introduced only at the onset of Stage 3 (after December 19, 2022). Consequently, raw comparisons of positivity rates between the two methods across the entire study period are invalid, as these differences are inextricably linked to temporal variations in virus prevalence, population immunity, and public health policies rather than assay performance alone. To mitigate this confounding, all valid comparisons between methods were restricted to Stage 3, where both assays were employed concurrently.

Within this overlapping period, it is imperative to accurately characterize the performance of each assay and the context of their application. Rapid antigen tests (RATs), such as the colloidal gold method employed herein, are well‐established to possess high specificity (> 95%) but moderate sensitivity (typically 50%–70% in clinical settings), often resulting in false negatives during periods of low viral load [20, 21]. Therefore, the observation that colloidal gold yielded higher positivity rates than PCR during Stage 3 is counter‐intuitive if attributed solely to intrinsic assay kinetics. This paradox is more plausibly explained by methodological biases rather than true differences in detection capability. First, clinical triage protocols dictated differential test allocation: colloidal gold was preferentially deployed for rapid screening in outpatients presenting with classic influenza‐like illness (high pre‐test probability), whereas PCR was reserved for hospitalized or clinically complex patients requiring broader differential diagnoses (who may have had lower pre‐test probability or co‐infections affecting viral shedding dynamics). Consequently, the higher TPR observed in the colloidal gold cohort likely reflects the selection of a higher pre‐test probability population rather than an inherent assay superiority. These selection biases necessitate cautious interpretation of apparent method‐specific differences in TPR [22, 23].

The stage of the pandemic significantly impacted positivity rates within the colloidal gold cohort. Compared to Stage 1, Stage 2 was associated with substantially decreased odds of positivity (OR = 0.35, 95% CI: 0.32–0.40), whereas Stage 3 saw markedly increased odds (OR = 2.22, 95% CI: 2.07–2.38). The use of colloidal gold testing during periods of heightened virus activity likely contributed to the observed surge in positivity rates; however, the interpretation of these estimates must account for the methodological shift. This highlights the need for methodological consistency and transparency in epidemiological studies to ensure reliable monitoring [24]. Moreover, the potential for misinterpreting viral spread emphasizes the importance of balancing speed and accessibility with accuracy, particularly for public health decision‐making [25].

The temporal analysis of TPR revealed significant fluctuations aligning with known patterns of influenza activity [26]. The sharp increase in positivity rates during 2023, as observed in both yearly and monthly data, suggests a potential outbreak or heightened virus activity during that period. However, this observation should be framed as an indicator of stress on the healthcare system and changes in the care‐seeking population, rather than a definitive measure of community attack rates. This surge coincided with the broader post‐pandemic phase, where relaxation of public health measures likely facilitated increased transmission, but also where changes in testing practices could have influenced the observed rates.

These findings highlight the utility of hospital‐based TPR for surveillance but also underscore the need for cautious interpretation. The observed variability in TPR across stages and demographics emphasizes that TPR is a proxy measure influenced by both viral dynamics and healthcare system factors. Vaccination remains a cornerstone of influenza prevention [16, 25, 27], however, its effectiveness can be influenced by factors such as antigenic match between the vaccine and circulating strains [28], as well as demographic characteristics like age and underlying health conditions [29]. The study underscores the importance of ongoing surveillance to monitor these dynamics and adjust public health responses accordingly. The marked increase in IAV positivity in 2023 aligns with the global post‐NPI respiratory virus rebound. This surge is consistent with the “immunity debt” hypothesis, wherein reduced viral circulation during stringent containment periods expanded the susceptible pool, culminating in widespread resurgence upon NPI relaxation. Furthermore, this period coincided with notable ecological shifts, including the apparent global cessation of influenza B/Yamagata lineage circulation. Thus, the observed spike is consistent with contemporaneous international reports, reflecting a broader trend in viral dynamics rather than a localized anomaly [30].

Despite the comprehensive nature of this study, several limitations must be acknowledged. First, the reliance on data from a single hospital limits the generalizability of the findings to other regions or populations. Second, potential confounders such as comorbidities, immune status, and prior vaccination history were not fully accounted for. The absence of individual‐level vaccination records—while contextual data indicate influenza vaccine coverage in mainland China‐including Mianyang‐remains low (< 5% in most adult cohorts) throughout the study period [31]. This low baseline strengthens our interpretation by suggesting that the observed 2023 IAV rebound was unlikely driven by vaccine failure, but rather by immunity debt and relaxed NPIs. Third, as a hospital‐based TPR analysis, the findings are subject to biases from temporal changes in testing volume and clinical thresholds, and healthcare‐seeking behaviors. The complete confounding between testing methodology and pandemic stage, coupled with selection bias from clinical triage in Stage 3, further limits causal inference regarding assay performance. This over‐representation is driven by a lower clinical threshold for testing febrile infants compared to adults. Consequently, the observed positivity rate in this youngest age group may be diluted by the inclusion of children with non‐influenza febrile illnesses (e.g., RSV, adenovirus, or other viral infections). This limits the generalizability of our age‐stratified positivity rates as direct measures of true population susceptibility or attack rates. Fifth, our assays detected Influenza A generically, and the inability to distinguish between H1N1pdm09 and H3N2 subtypes limits our understanding of clade‐specific dynamics and their contribution to the observed post‐pandemic rebound. Future studies employing multiplex PCR platforms are needed to elucidate these evolutionary patterns. Finally, the unit of analysis was the test record rather than the individual patient. Because the LIS extract contained no patient‐level identifier, repeat tests from the same individual could not be identified or removed, and the extent of such repetition was not quantified; consequently, some individuals may have contributed more than one record. This unaccounted within‐person clustering means that the 95% confidence intervals in Tables 2 and 3—and the corresponding p‐values—are likely narrower than they would be under a patient‐level analysis, and should be interpreted with caution. Meanwhile, future multi‐center studies incorporating detailed individual‐level covariates, particularly vaccination status, alongside subtype‐specific assays capable of discriminating between H1N1pdm09 and H3N2, and employing standardized protocols across all phases, are warranted to validate these findings.

## 5 Conclusions

In conclusion, this study characterizes the dynamics of hospital‐based influenza A virus (IAV) test positivity rates (TPR) across the pre‐, during‐, and post‐pandemic periods, revealing how pandemic stage, testing methodology, and healthcare‐seeking behavior jointly shape the observed patterns. TPR is a composite metric, driven by both viral circulation and healthcare‐system factors—notably temporal changes in testing volume and clinical triage thresholds. Because assay type was completely confounded with pandemic stage, and because Stage 3 was further shaped by selection bias from clinical triage, these limitations necessitate cautious interpretation: TPR is not a direct measure of community prevalence, but rather an indicator of healthcare system stress and symptomatic‐infection trends within the care‐seeking population. Moving forward, surveillance systems should prioritize methodological transparency and explicitly acknowledge the inherent biases of facility‐based data. Stage‐specific TPR monitoring offers a pragmatic basis for optimizing resource allocation and informing evidence‐based responses to influenza epidemics in the post‐pandemic era.

## Author Contributions

**Hao Dang:** investigation, conceptualization, methodology, validation, writing – original draft. **Xiujuan Peng:** methodology, validation, investigation. **Li Xu:** validation, investigation. **Wei Wang:** methodology, validation. **Ping Tang:** conceptualization, methodology, funding acquisition, writing – original draft, writing – review and editing.

## Ethics Statement

The clinical study was reviewed and approved by the Ethics Committee of the Third Hospital of Mianyang (The Affiliated Mianyang Hospital of Chongqing Medical University) (No. 2018‐IRB‐50). Waiver of informed consent for retrospective analysis of laboratory records.

## Conflicts of Interest

The authors declare no conflicts of interest.

## Data Availability

Data will be made available on request.
